# Dialysis access-associated steal syndrome with percutaneous endovascular arteriovenous fistula creation

**DOI:** 10.1186/s42155-022-00289-z

**Published:** 2022-02-26

**Authors:** Rakesh Varma, Manuel Betancourt-Torres, Eric Bready, Alian Al-Balas

**Affiliations:** 1 Division of Interventional Radiology, Department of Radiology, University of Alabama at Birmingham, 619 19th Street South, New Hillman Building, NHB-H623, AL 35249 Birmingham, USA; 2 Division of Nephrology, University of Alabama at Birmingham, 728 Richard Arrington Jr BLVD South, 35233 Birmingham, AL USA

**Keywords:** Steal syndrome, EndoAVF creation, WavelinQ

## Abstract

**Background:**

Dialysis access-associated steal syndrome (DASS) is an infrequent complication after hemodialysis access creation. Clinical symptoms depend on the degree of steal. Percutaneous arteriovenous fistula creation offers a minimally invasive alternative to surgical creation, though complications have been reported. The following presents the first described case of DASS after percutaneous endovascular arteriovenous fistula creation, and discusses risk factors and management.

**Case Presentation:**

Our case is that of a 27-year-old male with end stage renal disease due to congenital renal dysplasia, who underwent left percutaneous arteriovenous fistula creation for initiation of dialysis. Two months after the procedure the patient complained of coldness, pain, tingling, and numbness in the left arm during dialysis, concerning for steal syndrome. The patient subsequently underwent brachial artery angiogram, which showed minimal antegrade flow through the ulnar and interosseous arteries towards the hand, and a focal, severe stenosis in the distal ulnar artery. Angioplasty of the stenosis was performed, though steal symptoms continued.

**Conclusions:**

DASS, though rare, can be seen with percutaneous arteriovenous fistula creation. Identification of the risk factors prior to creation can help avoid this complication. Management is largely guided by clinical presentation. As long as there is adequate collateral supply to the extremity, single vessel occlusion is not a contraindication to percutaneous arteriovenous fistula creation with the use of WavelinQ technology. Careful patient selection with pre-creation angiogram may reduce the risk of symptomatic steal.

## Background

Arteriovenous fistula (AVF) remains the preferred vascular access for patients with end stage renal disease (ESRD) on hemodialysis. Although physiological steal (reversed flow in the distal outflow artery used for creating AVF) is common, symptoms of ischemia are rare due to compensatory vasodilation and collateral vessels, maintaining adequate distal perfusion. The reported incidence of Dialysis access-associated steal syndrome (DASS) in surgical AVF (sAVF) is 1-8% [1], though there have been no published case reports of DASS following percutaneous arteriovenous fistula (pAVF) creation. One case of DASS after pAVF creation mentioned in the NEAT trial [2] was the result of closure device mal-deployment within the brachial artery access leading to iatrogenically induced steal syndrome.

## Case presentation

**Fig. 1:**
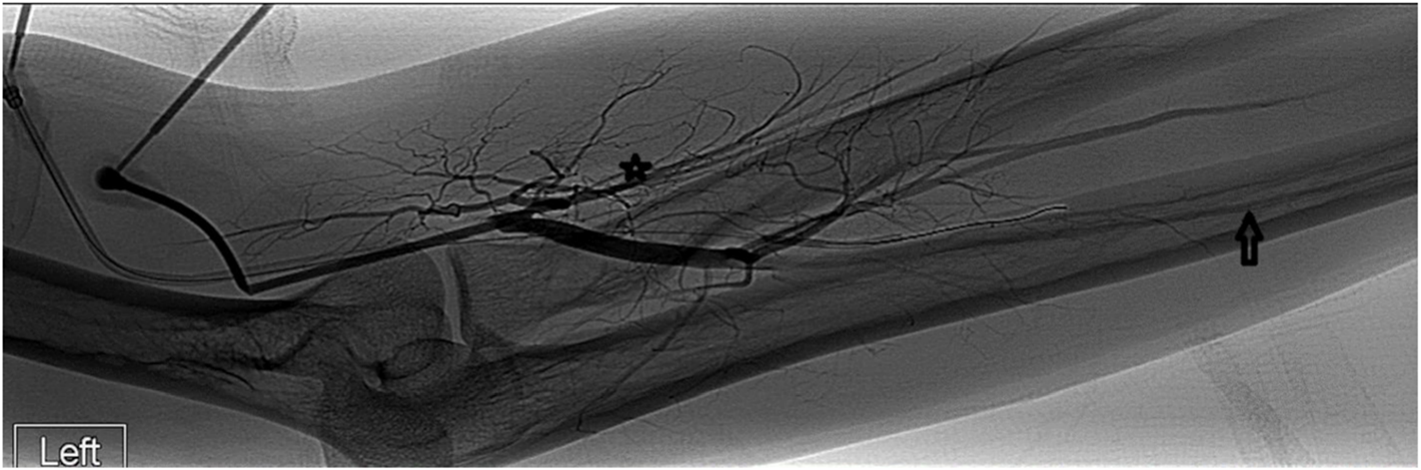
Pre-creation left brachial arteriogram in a 27-year-old man with ESRD. The radial artery (star) is diminutive in its proximal aspect and occluded in the mid forearm with flow in the hand from the ulnar artery (arrow) & interosseous artery

**Fig. 2:**
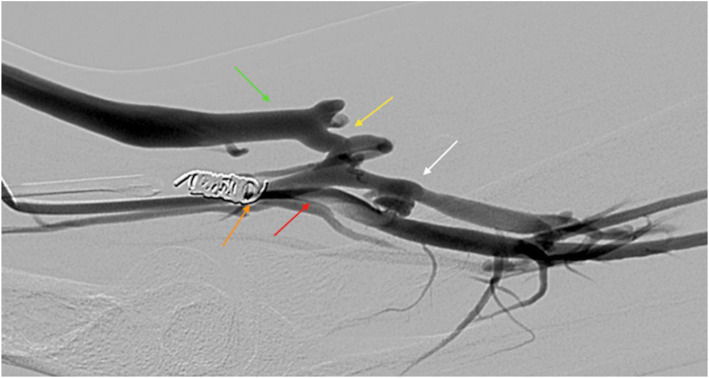
Post pAVF creation image using the WavelinQ technology in a 27-year-old man with ESRD. Arteriogram via the left arm brachial artery (red arrow) outlines the fistula (white arrow) between the ulnar artery & lateral ulnar vein with the perforator (yellow arrow) draining into the cephalic vein (green arrow). The accessed brachial vein was embolized with coils (orange arrow) for flow diversion into the superficial venous system

**Fig. 3:**
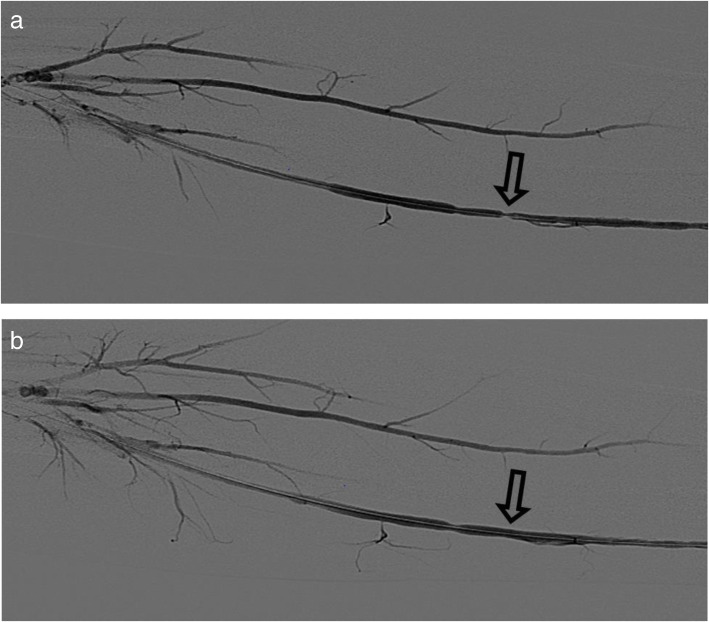
Left brachial angiogram in a 27-year-old man with ESRD s/p pAVF creation. Pre angioplasty (top image) demonstrates severe stenosis in the mid ulnar artery (open arrow). Post angioplasty (bottom image) demonstrates improvement in the stenosis.Earlier opacification of the ulnar artery and significantly improved flow in the interosseous and the radial artery was also seen (not shown), suggesting decreased resistance within the arterial bed post angioplasty

A 27-year-old right-handed male with ESRD secondary to congenital renal dysplasia presented for left forearm pAVF creation after meeting ultrasound mapping criteria for pAVF between the ulnar artery and vein. He had a prior failed left wrist radiocephalic AVF (rcAVF) secondary to arterial thrombosis. The radial artery was patent at the wrist but with diminutive flow sonographically. Precreation angiography confirmed a suitable location for fistula creation, in addition to chronic occlusion in the mid to distal segment of the radial artery (Fig. 1). The interosseous and ulnar arteries were patent, without ulnar artery stenosis identified. A pAVF was created (Fig. 2) using the 4 F WavelinQ (BD, Murray Hill, NJ) endovascular AVF (endoAVF) system with predominant flow via a perforator into the cephalic vein. The access lateral brachial vein was embolized with coils to promote flow through the superficial veins. Using Doppler ultrasound, established creation criteria for physiological maturation was met two weeks after creation and the fistula was cannulated using two needles for hemodialysis. Two months after the initial procedure the patient complained of coldness, pain, tingling and numbness in the left hand during dialysis, concerning for steal syndrome. Brachial angiogram demonstrated predominant flow through the fistula into the cephalic vein and minimal antegrade flow through the ulnar and interosseous arteries towards the hand. The distal ulnar artery showed a focal area of severe stenosis (Fig. 3A) and was the only vessel supplying the superficial palmar arch. On retrospective review, there was no retrograde filling of the radial artery at the wrist on the angiogram. Angioplasty of the ulnar artery stenosis was performed to 3 mm with near complete resolution of the stenosis (Fig. 3B). However, the patient continued to have steal symptoms during subsequent hemodialysis sessions. A multidisciplinary decision was made to continue using the pAVF as the symptoms only occurred during dialysis. The patient continues to be successfully dialyzed through the existing pAVF now for more than a year with mild steal symptoms.

## Conclusions

Risk factors for DASS include female gender, age > 60years, diabetes, atherosclerosis, previous access surgeries in the affected limb, and use of proximal versus distal artery for anastomosis [1]. The incidence of symptomatic steal is higher with a proximal creation site. Reported incidence is 1-2% with rcAVF versus 5-10% with brachial artery fistula.

Hand ischemia may occur during hemodialysis because dialysis tends to lower venous return, reducing cardiac output and lowering the perfusion pressure in the fistula outflow artery and collaterals that supply the hand. Acute presentation, i.e. occurring within hours of dialysis access creation, is more common with arteriovenous graft (AVG) and associated with poor vessel quality. Subacute and chronic presentations are usually associated with AVF. Symptoms can be mild including nail changes, occasional tingling and numbness during dialysis, or moderate to severe with muscle weakness, pale or cyanotic fingernail beds, rest pain, fingertip ulcerations and tissue loss [3]. Digital blood pressure, ultrasound and angiography can help with the diagnosis [3].

Four vascular beds that contribute to the pathophysiology of DASS are [1, 3]:

(i) Artery proximal to anastomosis: If diseased, cannot adapt to supply adequate flow to the arteriovenous access and distal extremity.
(ii) Artery distal to anastomosis: If diseased, can increase differences in resistance between vascular beds and result in DASS.
(iii) Draining veins: The larger the diameter of the draining vein the lower the resistance in the AVF, predisposing to DASS. Similarly, the diameter of the arteriovenous anastomosis can increase the risk.
(iv) Collateral arteries: Inadequate hypertrophy and dilation of the proximal and distal arterial system to supply adequate flow to the AVF and distal extremity can increase the risk of DASS.

DASS is also a factor in AVG, and is related to the larger diameter of the anastomosis relative to the artery. A smaller anastomotic diameter of 5 mm created by WavelinQ technology can also result in symptomatic steal [4].

Treatment is based on the severity of symptoms, and is often not required with mild DASS. Surgical management is indicated for moderate to severe symptoms. Distal Revascularization and Interval Ligation (DRIL), and Revision Using Distal Inflow (RUDI) are two surgical techniques that preserve the AVF and improve blood flow into the distal arm. Surgical ligation of the fistula is usually considered in patients with severe symptoms [3]. While the DRIL or RUDI surgical interventions may have improved our patient’s steal symptoms, these symptoms were mild, only occurring during dialysis, hence surgical intervention was not undertaken.

Our patient had an occluded distal radial artery and a focal area of severe stenosis in the distal ulnar artery, not seen at pre-AVF creation angiography, causing an increase in the resistance between the vascular beds, promoting DASS. Additionally, the angiogram demonstrated lack of sufficient collateral formation around the region of radial artery occlusion. The angiogram also showed a large diameter draining vein contributed by prior rcAVF creation, resulting in decreased vascular resistance and increased flow through the fistula. Thus our patient demonstrated 3 out of the 4 pathophysiological factors contributing to DASS.

DASS, though rare, can be seen with pAVF creation. Identification of the risk factors prior to creation, especially in ESRD patients who are at higher risk of peripheral vascular disease, can help avoid this complication. Management is largely guided by clinical presentation. As long as there is adequate collateral supply to the extremity, single vessel occlusion is not a contraindication to pAVF creation with the use of WavelinQ technology. Careful patient selection with pre-creation angiogram for pAVF may reduce the risk of symptomatic steal.

## Data Availability

Not applicable.
